# Supplementary material for: Electric‐Current‐Assisted Nucleation of Zero‐Field Hopfion Rings
Source: Adv Mater. 2026 Mar 17;38(21):e23417. doi: 10.1002/adma.202523417 (PMC13073127; doi:10.1002/adma.202523417)
Supplement: Supplementary file 1 — Supporting File 1: adma72762‐sup‐0001‐SuppMat.pdf. [file ADMA-38-e23417-s003.pdf]

Electric-current-assisted nucleation of zero-field hopfion rings
Supporting Information

Xiaowen Chen, Dongsheng Song, Filipp N. Rybakov,* Nikolai S. Kiselev,† Long Li, Wen Shi, Rui Wu, Xuewen Fu, Olle Eriksson, Stefan Blügel, Rafal E. Dunin-Borkowski, Haifeng Du, and Fengshan Zheng‡

* philipp.rybakov@physics.uu.se
† n.kiselev@fz-juelich.de
‡ zhengfs@scut.edu.cn

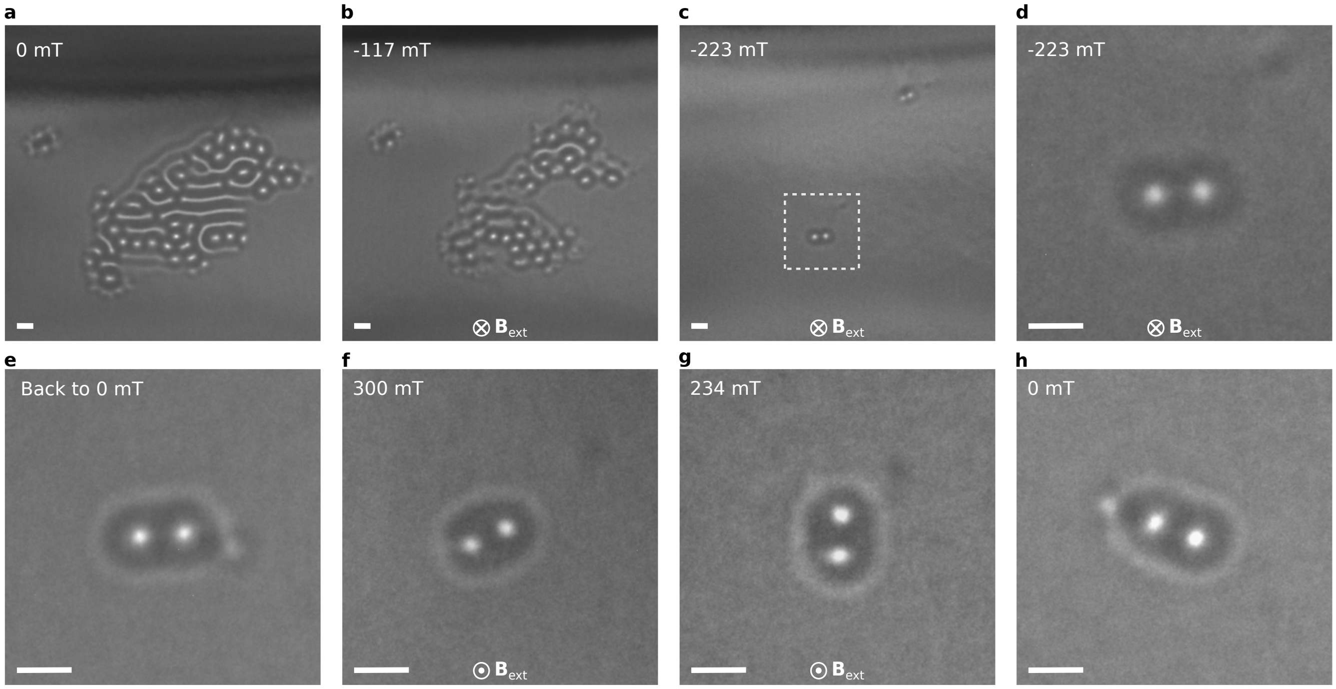

Supplementary Figure 1 | Hopfion ring nucleation. **a** illustrates the configuration that has been achieved by saturating the sample in the perpendicular magnetic field, subsequently reducing the external field to zero, and finally applying a short electric current pulse. **b** and **c** illustrate the evolution of the contrast under an external magnetic field applied in the negative direction – opposite to the saturating field. Positive and negative field directions correspond to the directions towards the viewer and away from the viewer as indicated by symbols $\otimes$ in **b-d** and $\odot$ in **f-g**. At this step, the large skyrmion clusters collapse, and we often end up with a few configurations with a hopfion ring as in **c**. **d** is a zoomed-in view of the image shown in **c** (dashed square). **e**, The contrast of the hopfion ring around two skyrmion strings after the magnetic field is reduced back to zero. The images **f** and **g** show the evolution of Lorentz TEM contrast as the field increases in the positive direction but nearly the same absolute value as in the negative direction in **c**. **h** displays the contrast after reducing the external magnetic field to zero again. It indicates that the hopfion ring remains stable even after a few cycles of applying positive and negative fields. The images were taken at a defocus distance of 700 μm and temperature 95 K. The scale bar in all images corresponds to 100 nm.

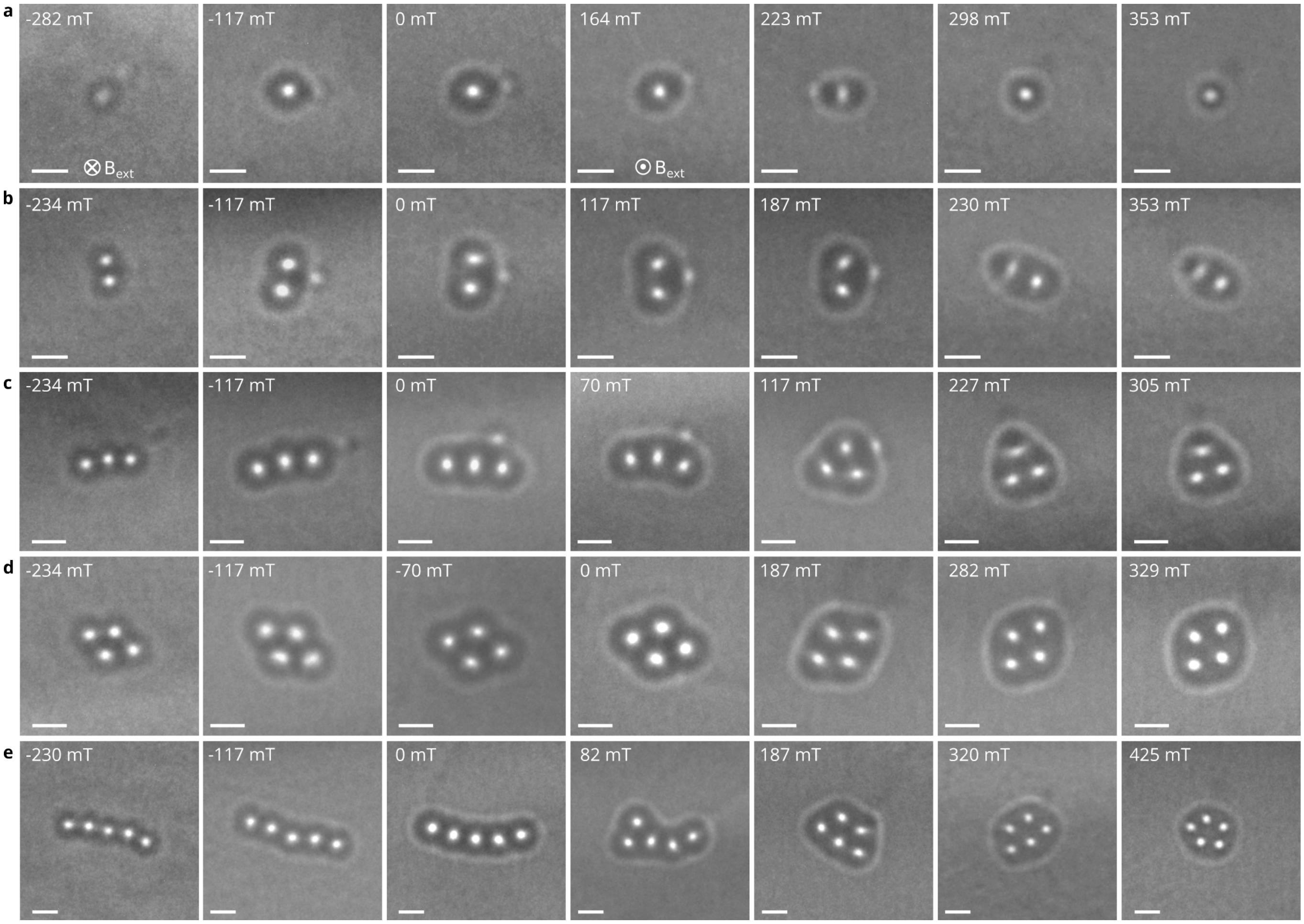

Supplementary Figure 2 | Field-driven evolution of hopfion rings. Each row of images represents the sequence of over-focus Lorentz TEM images recorded during the magnetic field reversal from a negative to a positive direction. The strength of magnetic field is labeled in the top-left of each images. Positive and negative field directions correspond to fields pointing toward and away from the viewer, as denoted by the symbols $\odot$ and $\otimes$ in **a**, respectively. In each row, the negative field decreases from left to right, reaches zero, and then increases in the positive direction. **a** and **b** are the extended versions of Figures **3a** and **b**, respectively. **d**, Extended version of Figure **2c**. Under negative fields, bumps are observed in the hopfion rings. As the positive field increases, these bumps gradually diminish and disappear at higher positive fields. The hopfion ring remains stable within this magnetic field range. The images were taken at a defocus distance of 700 μm and temperature 95 K. The scale bar in all images corresponds to 100 nm.

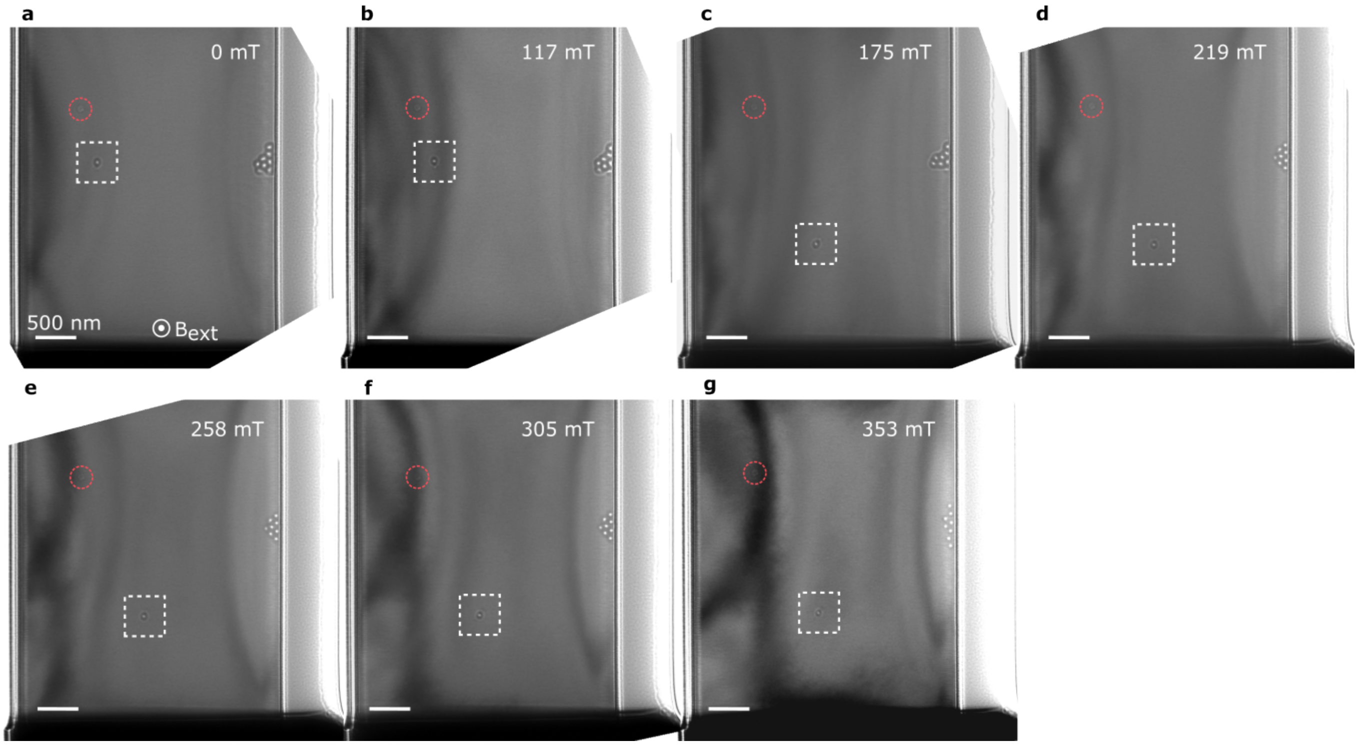

Supplementary Figure 3 | Field evolution of the hopfion ring. An extended version of Figure **3a**, showing the entire field of view of the state. Figure **3a** in the main text displays a zoomed-in region, marked here by a dashed rectangle and rotated clockwise by 90°. The texture movement is most pronounced in the field range between 117 mT and 175 mT. A red dotted circle marks a surface contamination spot, which serves as a reference point. All images were taken in an over-focus Fresnel TEM regime at a defocus distance of 700 μm and a sample temperature of 95 K. The scale bar in all images is 500 nm.

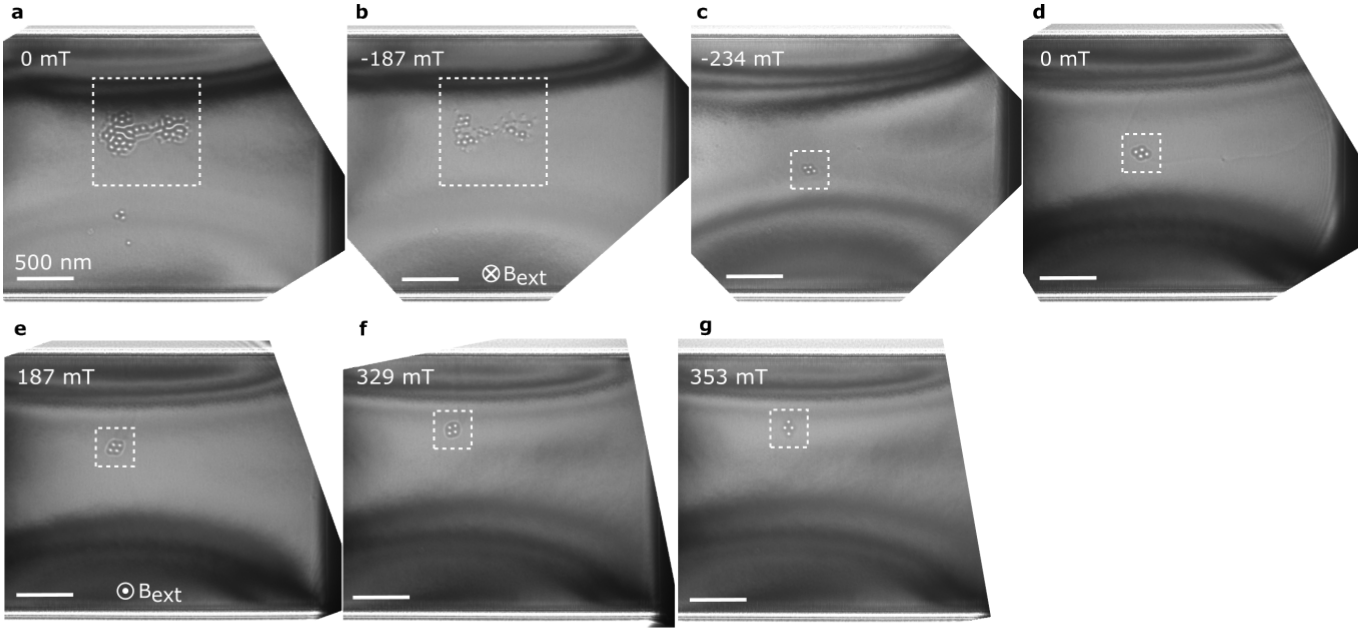

Supplementary Figure 4 | Field evolution of the hopfion ring. An extended version of Figure **2c**, showing the entire field of view of the state. The texture moves slightly during field-driven evolution. All images were taken in an over-focus Fresnel TEM regime at a defocus distance of 700 μm and a sample temperature of 95 K. The scale bar in all images is 500 nm.

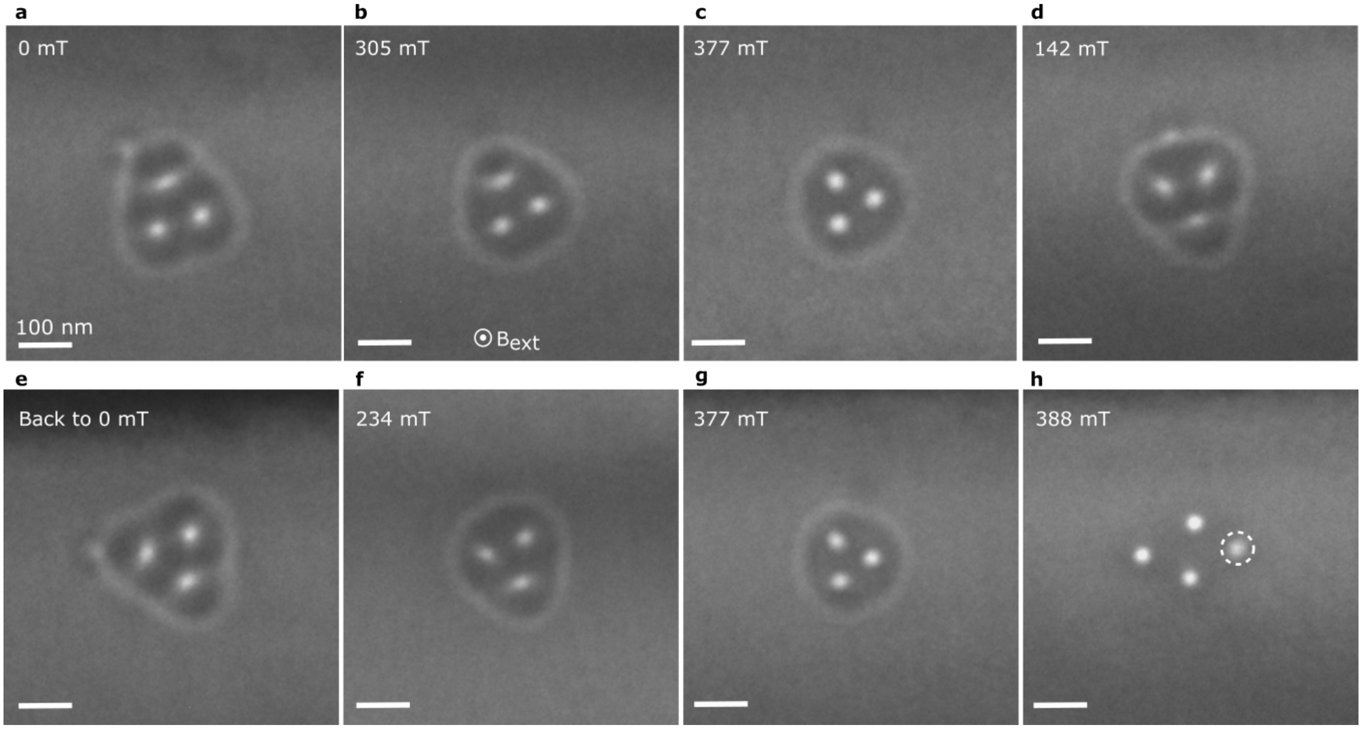

Supplementary Figure 5 | Field evolution of the hopfion ring. The images illustrate the emergence of a characteristic bump on the hopfion ring at low magnetic fields and its absence at higher fields. The dashed circle marks a low-intercity contrast spot associated with the chiral bobber or dipole string appearing after the collapse of the hopfion ring. All images were taken in an over-focus Fresnel TEM regime at a defocus distance of 700 μm and a sample temperature of 95 K. The scale bar in all images is 100 nm.

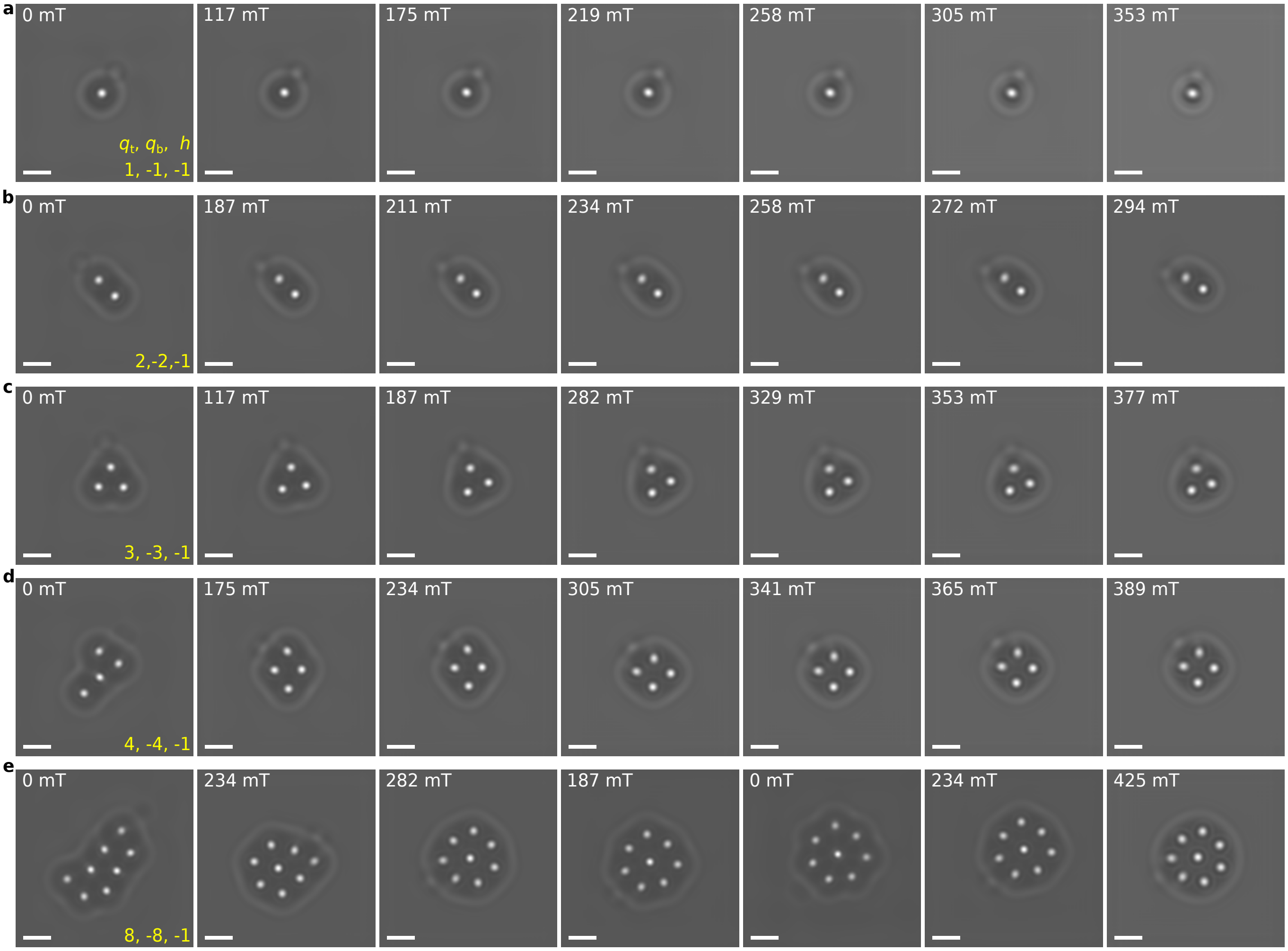

Supplementary Figure 6 | Field-driven evolution of hopfion rings in micromagnetic simulations. Each row of images represents the sequence of over-focus Lorentz TEM images calculated for magnetic configurations depicted in Figure **3** in the main text. Corresponding magnetic textures are obtained by energy minimization of the micromagnetic functional. The simulated domain has a size of 640 nm × 640 nm × 170 nm and periodic boundary conditions in the xy-plane (see Method for details). All images are calculated assuming a defocus distance of 700 μm. The scale bar in all images is 100 nm.

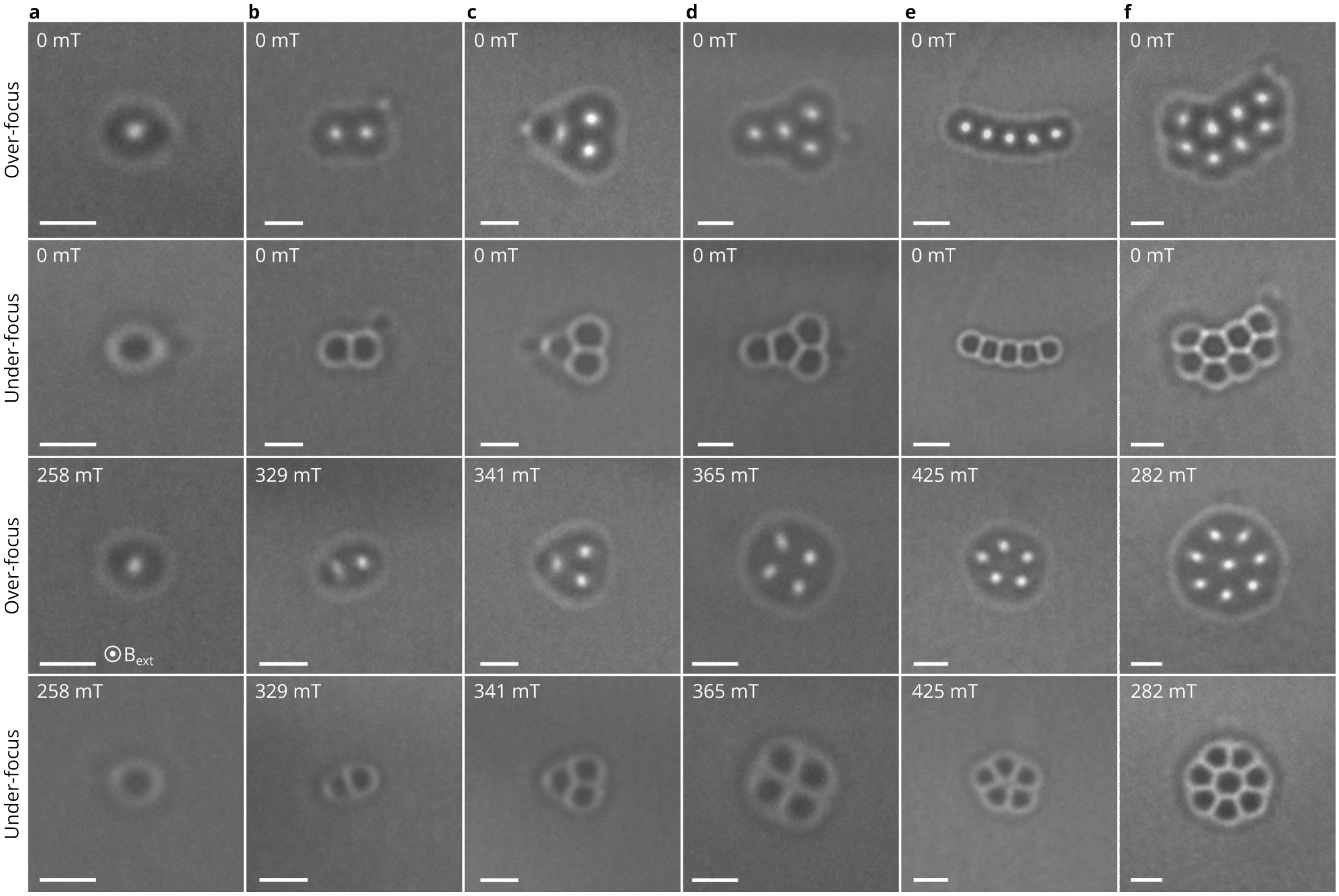

Supplementary Figure 7 | Collection of over-focus and under-focus Lorentz TEM images for representative configurations. Each column contains two pairs of Lorentz TEM images acquired in over-focus and under-focus conditions, illustrating the zero-field and high-field states of the same magnetic configuration. The magnitude of the applied magnetic field is indicated on the left side of each image. All images were recorded at the defocus distance of ± 700 μm, and the sample temperature was 95 K. The scale bar in all images is 100 nm.

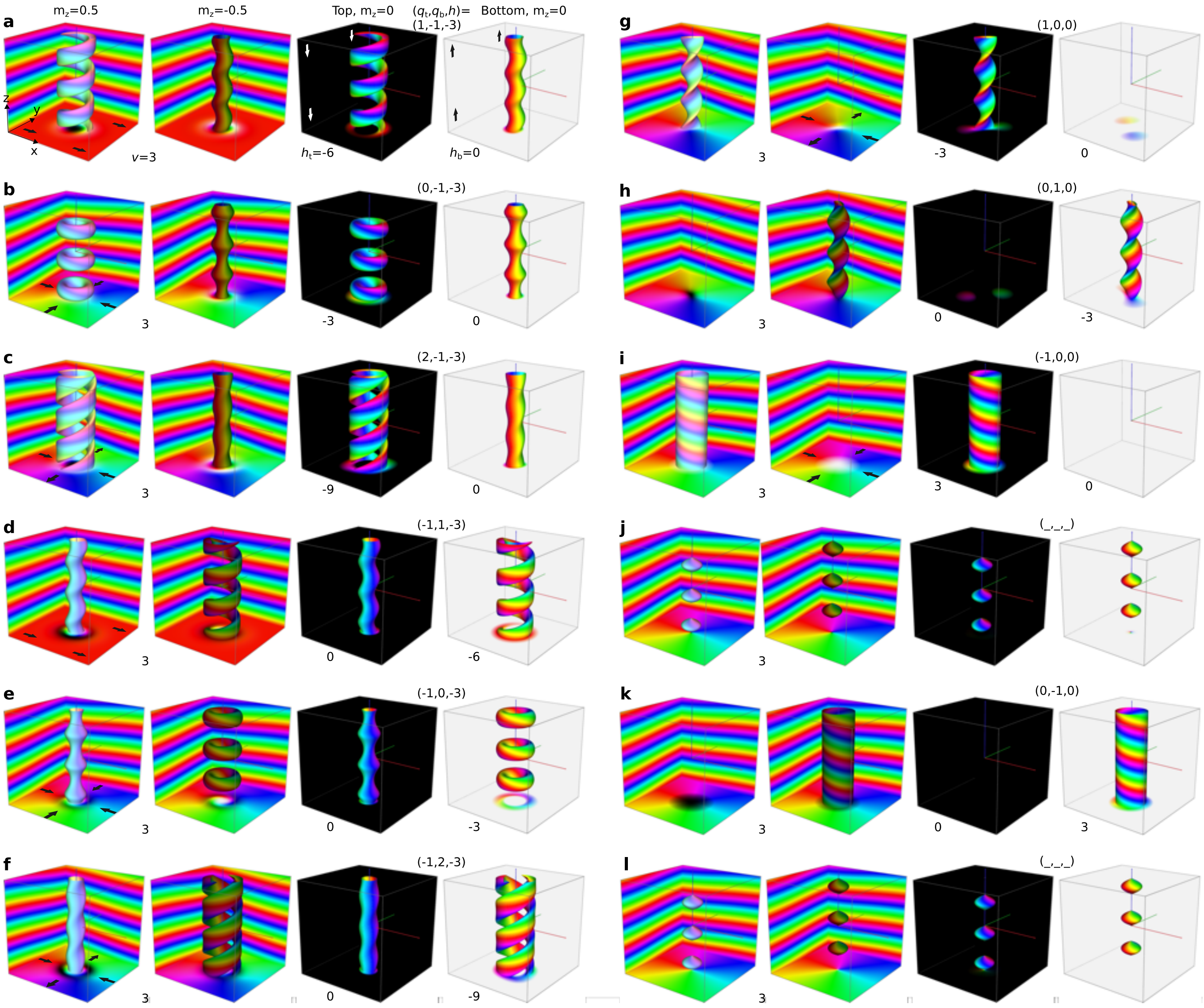

Supplementary Figure 8 | Collection of topological magnetic textures (skyrmions and vortices). This figure presents a set of magnetic configurations similar to Figure **5** in the main text but with a different selection of textures. Skyrmions in helical backgrounds: **a**, Skyrmion in a regular helical vacuum. **b**, Skyrmion embedded in a helical vortex. **c**, Skyrmion embedded in a helical antivortex. **d**, **e** and **f** Same as **a**, **b**, and **c** but with opposite 2D indices. Isolated helical vortices and antivortices: **g**, Isolated helical antivortex with its core polarity along the positive z-axis. **h**, Same as **g** but with opposite core polarity. **i**, Isolated helical vortex with its core polarity along the positive z-axis (unstable). **j**, The relaxed configuration obtained after energy minimization of the state shown in **i**. Such a configuration is known in the literature as *screw dislocation* of a particular type[33]. Since this configuration contains Bloch points (point singularities), the homotopy classification presented here is not applicable. **k**, **l** Same as **i** and **j** but for a helical vortex with opposite core polarity.

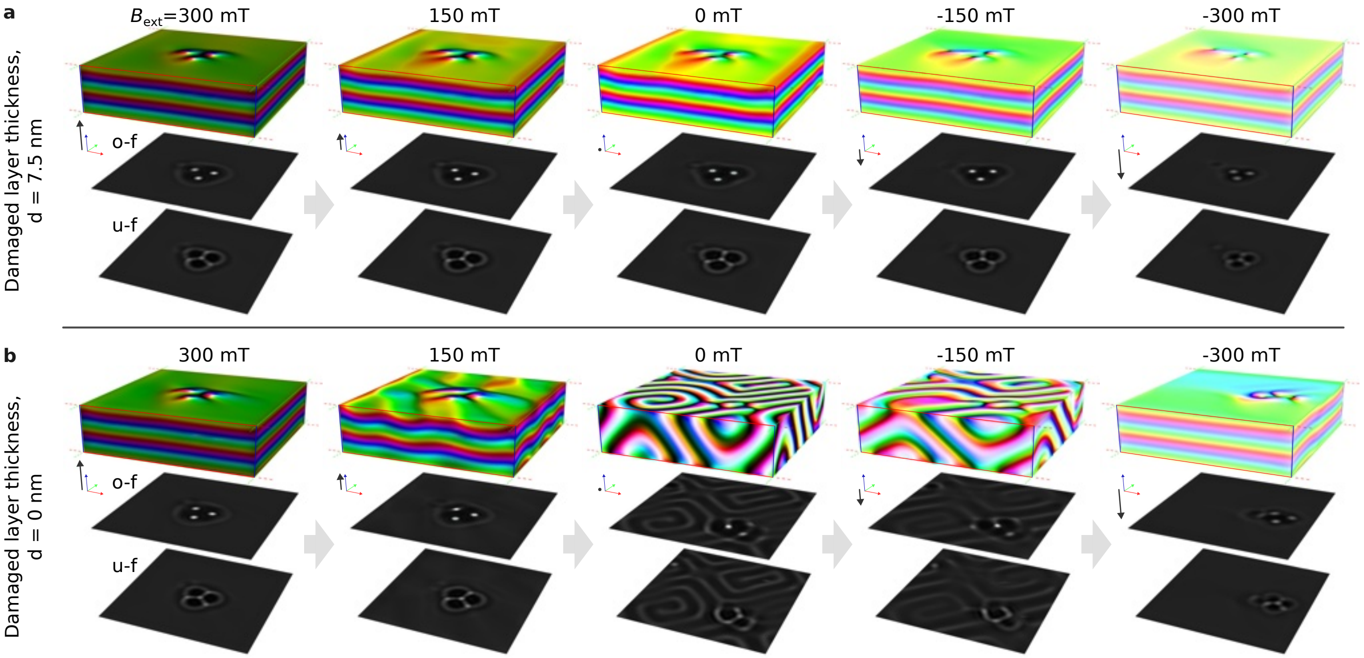

Supplementary Figure 9 | Effects of Surface Damaged Layer. The micromagnetic simulations were performed in a domain of size $640 \times 640 \times 170$ nm^3 (mesh density $256 \times 256 \times 68$ cuboids) and periodical boundary conditions in the xy-plane. The external magnetic field is applied parallel to the z-axis. In **a**, the thickness of the surface damaged layer on both top and bottom surfaces is 7.5 nm. For the damaged layer properties, see Methods. In **b**, we assume there are no damaged layers. Each image shows magnetization on the simulated box's surface, represented by the standard color code, and two over-focus (o-f) and under-focus (u-f) Lorentz TEM images on the top and bottom, respectively. In both cases, the states are obtained by sequentially reducing the field from 300 mT to -300 mT, as indicated by arrows. Note the sequence of states in **a** is fully reversible as in experimental observations, while the states depicted in **b** are not reversible. In the absence of the damaged layer, with a decreasing external magnetic field, the surface modulations penetrate the whole film thickness and form a labyrinth texture. The configurations depicted in **a** for three skyrmions and one hopfion ring are identical to those shown in Figure **2** in the main text. In contrast, micromagnetic simulations that exclude the damaged layer predict a contrast pattern that is not observed in our experiment.

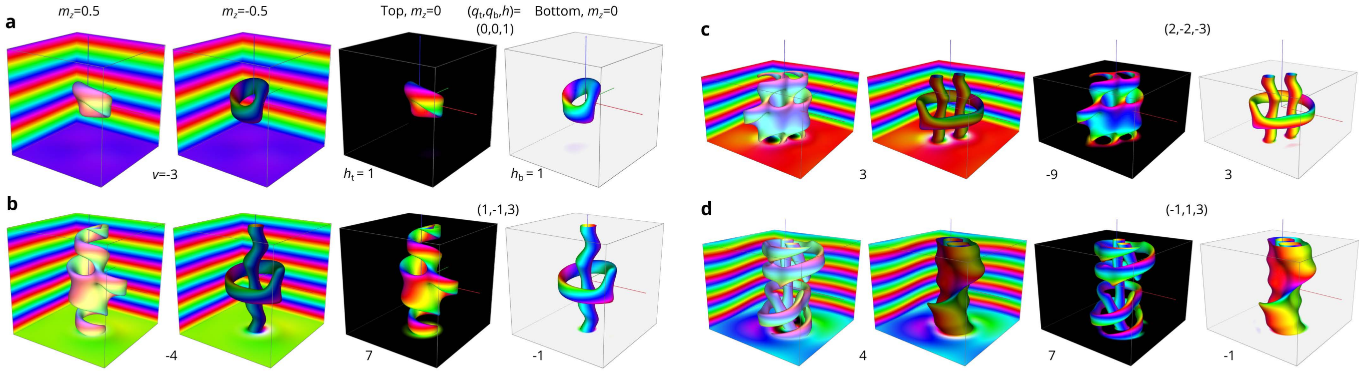

Supplementary Figure 10 | Collection of topological magnetic textures with different topological index (q_t, q_b, h)**.** This figure presents a set of magnetic configurations similar to Figure **5** in the main text and Figure **8** but for a different set of textures. **a** and **b**, are isolated hopfions embedded into a helix (heliknoton) and hopfion ring that are similar to those in Figures **5f** and **g**, respectively, but for the systems with opposite (left-handed) chirality. **c**, Single hopfion ring on a pair of non-braiding skyrmion strings. **d**, A hybrid skyrmion string, as described in Ref.[42].

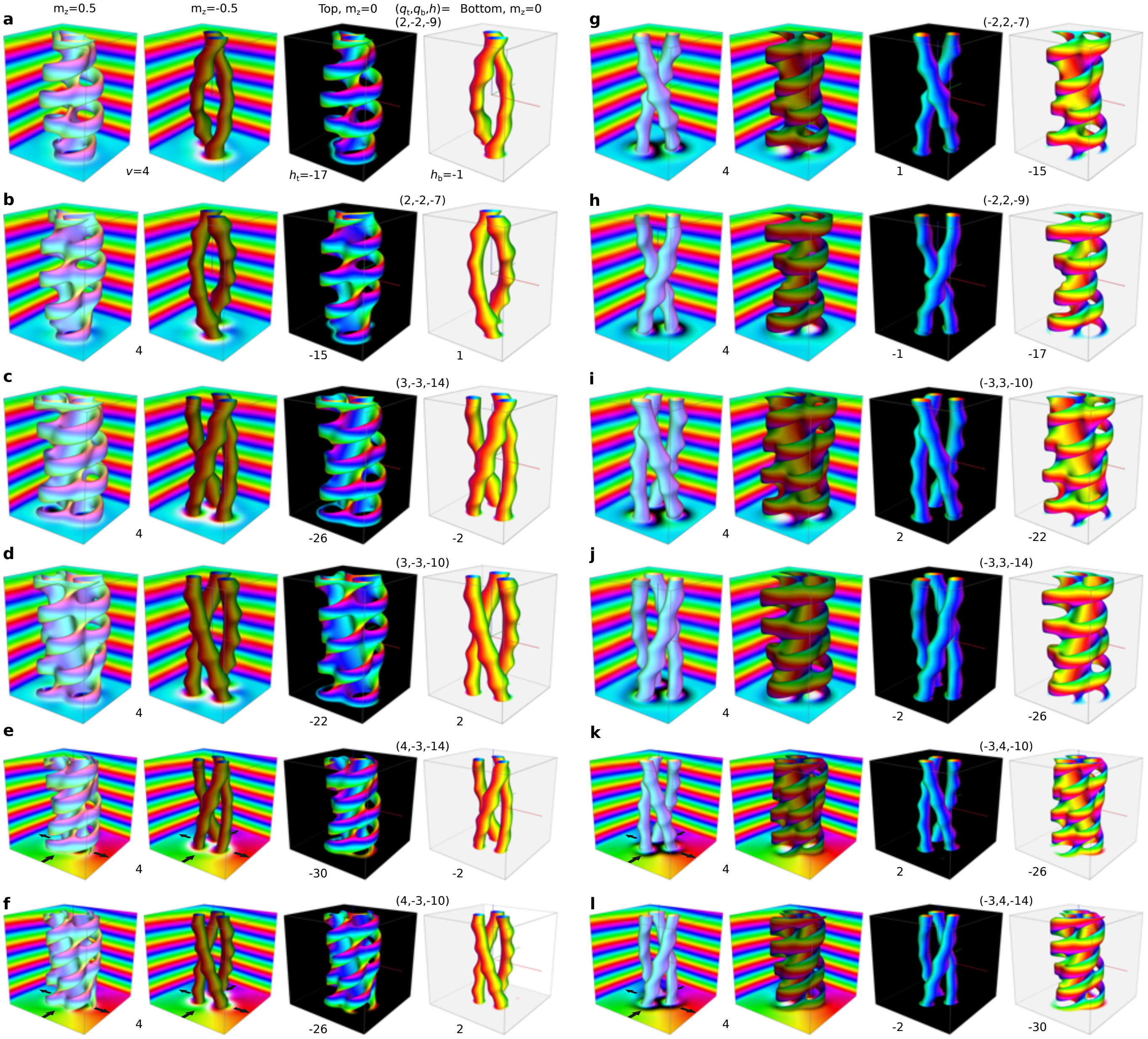

Supplementary Figure 11 | Collection of topological magnetic textures (skyrmion and antiskyrmion braids). This figure presents a set of configurations similar to to Figure **5** in the main text and Figure **8** but featuring various skyrmion braids with different numbers of skyrmion strings, skyrmion topological indices, and braid twisting angles. Notably, the skyrmion braids in **e**, **f**, **k**, and **l** are embedded in a helical antivortex, whereas the remaining configurations reside in a regular helical background.
